# Heat shock proteins in atrial fibrillation: from bench to bedside

**DOI:** 10.3389/fphys.2025.1644898

**Published:** 2025-07-25

**Authors:** Shujie Zhang, Yujie Wang, Lujing Nie, Wenjiu Feng, Mengyuan Zhang, Yanbo Chen

**Affiliations:** ^1^ The First Affiliated Hospital of Shandong Second Medical University, Weifang, China; ^2^ Changle County People’s Hospital, Weifang, China; ^3^ The Affiliated Hospital of Shandong University of Traditional Chinese Medicine, Weifang, China

**Keywords:** atrial fibrillation, heat shock proteins, inducer, proteostasis, inflammation, oxidative stress

## Abstract

Atrial fibrillation (AF) is the most common age-related arrhythmia in clinic, affecting millions of people around the world, and is closely related to heart failure, ischemic stroke and other diseases. In addition, AF is progressive in nature and represents a significant global health burden. However, the current treatment plans are mainly symptomatic, the efficacy in preventing atrial fibrillation is limited. Hence, there is a pressing need for etiology-specific AF treatments. It is widely acknowledged that the atrial electrical and structural remodeling constitutes the pathological basis of atrial fibrillation. Evidence indicates that heat shock proteins (HSPs) could have a protective effect against AF. HSPs are a diverse family of molecular chaperones that safeguard cells against various stressors. They play a crucial role in mitigating oxidative stress, inflammation, and apoptosis, thereby helping to prevent structural and electrical remodeling in cardiomyocytes. Moreover, HSPs safeguard proteostasis via prevention of toxic protein aggregation by binding to (partially) unfolded proteins. As pivotal inhibitors of AF onset and progression, HSPs represent both a promising therapeutic target and potential biomarkers for staging AF and predicting post-treatment recurrence, as evidenced by recent studies. In this review, we explore the mechanisms of HSP in AF to pave the way for the development of targeted therapies for this prevalent arrhythmia disease.

## 1 Introduction

AF is the most frequently observed arrhythmia in older adults in clinical settings, affecting 46 million people worldwide (Kornej et al., 2020). With the aggravation of population aging, the change of lifestyle and the progress of related detection technology, the prevalence and incidence rate of AF are still rising year by year. The clinical consequences of atrial fibrillation are particularly grave, as it significantly increases risks of stroke, heart failure, hospitalizations, degraded quality of life and reduced exercise capacity (Hindricks et al., 2021). Atrial fibrillation arises from complex interactions among various factors, and the exact mechanisms underlying its development remain unclear. At present, the treatment methods for AF mainly include drug therapy and catheter ablation, but these treatments are only moderately (Sirish et al., 2022). The therapeutic effectiveness of currently approved antiarrhythmic medications remains limited, with most agents carrying a significant risk of proarrhythmic effects, including potentially fatal ventricular tachyarrhythmias (van Gorp et al., 2020). The total success rate for first catheter ablation is about 56%, and the success rate of patients over 60 years old is significantly reduced (Ayzenberg et al., 2023). Therefore, there is an unmet need for effective method that detect and treat patients with atrial fibrillation.

Emerging evidence indicates that HSPs may exert protective effects against atrial fibrillation (Hazra et al., 2023). HSPs play significant roles in the pathogenesis of numerous diseases, encompassing cancer, neurodegenerative disorders, and autoimmune conditions (Singh et al., 2024). HSPs were first identified by Ferruccio Ritossa in *Drosophila melanogaster* in the 1960s (Currie, 1988). However, it wasn't until the 1980s that William Currie conducted in-depth research on heart tissue. Current evidence indicates that HSPs, as molecular chaperones, are involved in the pathogenesis of various diseases, including cancer, neurodegenerative disorders, and autoimmune diseases, among others. Based on differences in molecular weight and sequence similarity, HSPs are classified into seven HSP families, HSPA(HSP70), HSPB (small HSPs), HSPC (HSP90), HSPD/E (HSP60/HSP10), DNAJ (HSP40) and CCT, each with several family members (Kampinga et al., 2009). They involve in the protection against various forms of cellular stress. Protective effects of HSPs against oxidative stress and inflammation have been described, indicating their potential in preventing the occurrence of AF (Attia et al., 2025). Beyond that, HSPs can regulate protein folding, localization, degradation and function, thereby maintaining proteostasis and preventing the progress and maintenance of AF (Li and Brundel, 2020; Henning and Brundel, 2017). Moreover, some studies have shown that HSPs may be used as biomarkers to discriminate between the various stages of AF and recurrence of AF after treatment.

In this review, we summarize current evidence on the role of HSPs in the pathogenesis and progression of AF. We further explore their potential clinical applications, focusing on HSPs as predictive biomarkers and the therapeutic potential of HSP-inducing compounds in AF management.

## 2 Mechanism of occurrence and progression of AF

The pathogenesis of AF is complex and multifactorial. It is currently believed that various factors interact to cause structural and electrophysiological changes in the atria, leading to the development and progression of AF (Hu et al., 2023). While it is likely that multiple mechanisms contribute to AF risk, inflammation and oxidative stress seem to play large roles (Gao and Dudley, 2009).

### 2.1 Inflammation promotes the occurrence and maintenance of atrial fibrillation

A plethora of evidence showed that AF is associated with inflammatory process. A study showed that the increased AF incidence, as observed in endurance exercise mouse models (swimming or treadmill-running), is associated with increased inflammation and TNFα-dependent activation of NFκB (nuclear factor kappa-light-chain-enhancer of activated B cells) in atrial cardiomocytes (Aschar-Sobbi et al., 2015). TNF-α is an inflammatory mediator associated with atrial fibrillation (Lakin et al., 2023). The action of TNFα is mediated through its receptors TNFR (TNFα receptor) located on immune and nonimmune cells. Besides this, it has been shown that activation of TNFα signaling can promote atrial electrical, structural, and contractile remodeling (Ren et al., 2015). This may be due to that inflammatory cytokines can cause adverse remodeling in cardiomyocytes and enhance AF susceptibility (Scott et al., 2019). NF-κB regulates the transcription of NOD-like receptor family pyrin domain-containing 3(NLRP3) (He et al., 2016),which directly lead to AF. In turn, once AF occurs, it will induce local and systemic inflammation, making AF easy to sustain (Ihara and Sasano, 2022). There is a complex bidirectional relationship between inflammation and AF, where the two promote each other through multiple mechanisms, forming a vicious cycle (Vyas et al., 2020). This suggests that future AF treatment should integrate rhythm control and anti-inflammatory strategies by implementing personalized inflammation-targeted interventions.

### 2.2 Oxidative stress contributes to the risk of atrial fibrillation

**FIGURE 1:**
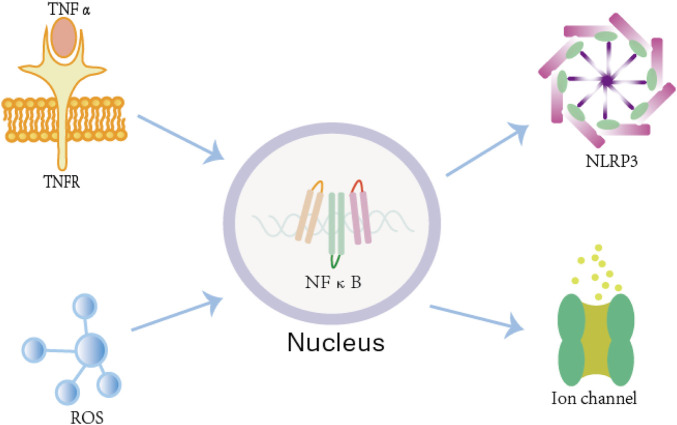
The mechanism by which inflammation and oxidative stress lead to atrial fibrillation. Both the TNF-α/TNFR complex and the ROS system can activate NF-κB in atrial cardiomyocytes. Once activated, NF-κB upregulates NLRP3 inflammasome expression, which directly contributes to the development of AF. Additionally, NF-κB can directly modulate ion channel gene expression by binding to their promoter regions, further influencing atrial electrical and structural remodeling.

There is evidence that systemic and cardiac oxidative stress may contribute to the risk of AF (Gao and Dudley, 2009). Oxidative stress is defined as an increase in intracellular ROS such as H_2_O_2_, superoxide (NO^2-^), or hydroxyl radical (•OH) (Masuda et al., 2024). ROS are major activators of NF-kB (Zakkar et al., 2015). NF-κB can directly modulate ion channel gene expression by binding to promoter regions, regulate the expression of other transcription factors, and influence mRNA splicing patterns (Masuda et al., 2024). In turn, these three mechanisms collectively enhance ROS production, which activates NF-κB signaling and ultimately promotes atrial fibrillation development (Figure 1).

### 2.3 Derailment of proteostasis leads to atrial fibrillation

Beyond the well-established roles of inflammation and oxidative stress, accumulating evidence indicates that age-related proteostasis decline represents another critical pathway contributing to atrial fibrillation pathogenesis (Pandey et al., 2025). Due to the highly differentiated nature of cardiomyocytes, protein homeostasis is particularly crucial for aging myocardial cells (Zhang et al., 2017). Derailment of proteostasis is one of the root cause of AF promotion (Attia et al., 2025). Current evidence implicates three primary mechanisms of proteostasis impairment in the progression of atrial remodeling and AF development, including impaired protein quality control (PQC) system, histone deacetylase 6 (HDAC6)-induced disruption of the microtubule network, and DNA damage-induced PARP-1 activation with subsequent depletion of NAD + levels in mitochondria. A disruption in proteostasis activates the heat shock response (HSR), which in turn stimulates the production of HSPs. Specifically, HSPs serve as the core components of the PQC system. They function as molecular chaperones, aiding in protein folding and repair. If repair is unsuccessful, HSPs mediate the targeted degradation of the damaged proteins (Romanucci and Della Salda, 2015). The PQC system is capable of specifically identifying misfolded, orphaned, and mislocalized proteins. It then precisely directs these aberrant proteins to distinct degradation pathways. In particular, PQC facilitates selective protein degradation via either the ubiquitin-proteasome system (UPS) or the ESCRT-mediated multivesicular body-lysosome pathway (Kahlhofer et al., 2021; Yang et al., 2020). This intricate quality control network dynamically regulates protein synthesis, folding, localization, and degradation, thereby effectively maintaining intracellular proteostasis (Schwabl and Teis, 2022). Based on this, HSP inducers (e.g., GGA-59) can accelerate the structural and functional recovery of cardiomyocytes, while NAD^+^ supplements (as demonstrated in clinical trials such as the HF-AF ENERGY trial) have shown efficacy in reducing AF burden (Kishore et al., 2023).

## 3 Potential protective role of HSPs in AF

HSPs have been shown to be cardioprotective in a variety of paradigms (Shan et al., 2020). The HSP family exerts anti-AF effects through anti-inflammatory, antioxidant, and electrical stability maintenance mechanisms. Among all HSPs, HSPA (HSP70) and HSPB (HSP27) are the most widely researched. In the following section, a brief review of the HSPs is given.

### 3.1 Potential protective role of HSPA (HSP70) in AF

HSPA represents the gene family that encodes members of the 70-kDa heat shock protein (HSP70) family. Under oxidative stress, HSPA upregulation exerts cytoprotection by simultaneously suppressing NF-κB-mediated inflammation, maintaining proteostasis and inhibiting apoptosis. As a key regulator of inflammation, HSPA directly binds and sequesters NF-κB to suppress its signaling pathway, thereby suppressing the expression of inducible nitric oxide synthase (iNOS) (Szyller et al., 2022). This regulatory mechanism ultimately leads to a marked reduction in the production of reactive oxygen species (ROS) and peroxynitrite (Kumar et al., 2021). This anti-inflammatory mechanism complements its ability to interfere with TLR4-mediated inflammatory cascades triggered by DAMPs like hyperglycemia and oxidative stress, which normally activate NF-κB and promote pro-inflammatory cytokine release (TNF-α, IL-6, IL-1β) (Shi et al., 2023). Beyond its immunomodulatory functions, HSPA serves as a crucial molecular chaperone that maintains cellular proteostasis by facilitating protein (re)folding, mediating transmembrane transport for organelle-specific delivery, recruiting damaged proteins to proteasomes for degradation, and directing protein cargo to autophagic pathways (Bellini et al., 2017). By performing these multifaceted roles, HSPA suppresses protein misfolding and aggregation, thereby ensuring the preservation of proteostasis. Once the function of proteostasis network declines, cells will exhibit impaired folding efficiency for nascent polypeptides and diminished stability of metastable proteins (Tashiro et al., 2018). Under cellular stress conditions, this functional deterioration of protein conformations becomes particularly pronounced, resulting in substantial loss of biologically competent protein structures. With respect to apoptosis inhibition, HSPA binds to Apaf-1, preventing its recruitment of caspase-9 to form the apoptosome, thereby acting as a critical brake at the upstream level of the (mitochondrial) intrinsic apoptotic pathway (Wei et al., 2024).

Variations in HSPA expression levels demonstrate strong clinical correlations. Kornej et al. (2013) and colleagues conducted a prospective study involving 67 AF patients to evaluate the association between HSP70 levels and catheter ablation outcomes. Using enzyme-linked immunosorbent assay (ELISA), the researchers measured serum HSP70 and anti-HSP70 antibody concentrations at baseline and post-ablation. Key findings demonstrated that persistent AF patients exhibited significantly higher baseline anti-HSP70 antibody levels (median 53 μg/mL, IQR 41–85) compared to paroxysmal AF cases (median 43 μg/mL, IQR 28–62; p = 0.035). Furthermore, the study documented a marked elevation in both HSP70 protein and corresponding antibody titers following the ablation procedure. Notebly, only intracellular HSP70 elevation (not serum) post-ablation predicted reduced AF recurrence (p < 0.01), indicating compartment-specific cardioprotection (Mandal et al., 2005). The investigators concluded that these increases showed significant correlations with three clinical parameters: total ablation energy delivery (r = 0.42, p < 0.01), procedural duration (r = 0.38, p = 0.02), and importantly, the risk of AF recurrence during follow-up (HR 1.45, 95% CI 1.12–1.88) (Kornej et al., 2013). Notably, the protective role of HSP70 may extend beyond ablation outcomes. Mikel Allende’s (Allende et al., 2016) study further revealed an unexpected link between HSP70 expression and a reduced risk of cardioembolic stroke in AF patients. Importantly, HSP70 appears to play a protective role by inhibiting thrombus formation without increasing bleeding risk. Pharmacological induction of HSP70—using agents such as TRC051384 or tubastatin A—could offer a promising therapeutic strategy for AF patients requiring safe, long-term anticoagulation (Allende et al., 2016).

The HSP70 family is extensive, encompassing not only HSP70 itself but also members such as HSPA1, HSPA5, and HSPA9, each exhibiting distinct biological functions. HSPA5 alleviates ER stress and regulates calcium homeostasis (Schäuble et al., 2012; Wang et al., 2017); HSPA9 upregulated over twofold in atrial fibrillation, maintains mitochondrial proteostasis (Kirmanoglou et al., 2004); while HSPA1 induction delays thrombosis without affecting bleeding parameters, offering potential therapeutic advantages over traditional anticoagulants (Allende et al., 2016).

### 3.2 Potential protective role of HSPB (small HSP) in AF

Small HSPs are a group of low-molecular-weight HSPs in the range of 12–43 kDa and are classified as HSPB (Yamada et al., 2021). Cardiomyocytes highly express several specific members of the small HSP family, including HSPB1 (HSP27), HSPB6 (HSP20), HSPB7 (cvHSP), and HSPB8 (HSP22) (Ke et al., 2011). HSPB induction preserves cardiac contractile function during tachypacing by maintaining calcium homeostasis and stabilizing microtubule structure (Zhang et al., 2011).

The most prominent heat-inducible cytosolic member of the human small HSP family is known as HSPB1. To molecularly demonstrate the role of HSP27, Brundel et al. (2006a). conducted both *in vivo* and *in vitro* experiments using canine models. Their results indicate that phosphorylation-dependent HSP induction maintains Ca^2+^ handling and contractile function in tachypaced myocytes, as evidenced by identical protection from a phosphomimetic HSP27 mutant. Hu et al. (2019) examined the function of HSP at the organelle level. They demonstrated that HSPB1 safeguards microtubule integrity by binding to and inhibiting HDAC6, thereby maintaining α-tubulin acetylation and preventing microtubule disassembly and breakdown. Research in experimental and clinical AF revealed a role for microtubule disruption in AF promotion. The disruption of microtubule structure, influencing the derailment of proteostasis, impairs the contractile function of cardiomyocytes, thereby promoting the development of atrial fibrillation (Zhang et al., 2014). Futhermore, HSPB1 also reduces oxidative stress by increasing both glutathione levels and glyceraldehyde 3-phosphate dehydrogenase (GAPDH) activity (Leite et al., 2016).

HSP27 demonstrates significant clinical potential in practical applications. Immunohistochemical analysis demonstrated significantly elevated HSP27 expression exclusively in paroxysmal AF patients’ atrial tissue (p < 0.05 vs. persistent AF), suggesting its potential role in protecting cardiomyocytes from structural degradation and potentially delaying AF progression to persistent forms (Brundel et al., 2006b). Furthermore, HSP27 levels were significantly elevated in patients with post-PVI AF recurrence, suggesting its potential as a recurrence biomarker (Marion et al., 2020; Hu et al., 2012).

### 3.3 Potential protective role of HSPC (HSP90) in AF

The highly conserved HSPC/HSP90 family comprises 5 members, including HSPC1/HSP90AA1 (cytosol inducible), HSPC2/HSPAA2 (cytosol inducible), HSPC3/HSPAB1 (cytosol constitutive), HSPC4/GRP94 (ER) and HSPC5/TRAP1 (mitochondria) (Bellini et al., 2017). HSPC represents a double-edged sword in cardiovascular biology, exhibiting complex effects. The cardioprotective properties of HSPC are mediated through multiple mechanisms: it stabilizes cardiac ion channels (e.g., HERG) to modulate electrophysiology and prevent arrhythmias such as long QT syndrome (Iwai et al., 2013), while also binding to eNOS and suppresses Akt1-mediated phosphorylation of eNOS to prevent its aberrant translocation to mitochondria (Sun et al., 2024). Conversely, HSPC demonstrates pro-inflammatory and pro-fibrotic potential. By maintaining IKK and JAK2 stability, HSPC facilitates activation of both NF-κB and STAT pathways, thereby promoting inflammatory responses. Emerging evidence further suggests that HSPC inhibition may attenuate fibrotic processes, potentially through modulation of TGF-β1 signaling (Huang et al., 2025). This dual functionality underscores the necessity for cautious application.

In clinic practice, HSPC4 level was significantly increased in chronic AF. The increase in HSPC4 that occurs within cardiomyocytes was observed in an experimental model of AF in the goat and in human samples obtained from patients with chronic AF. This may be part of a cell protective program (Vitadello et al., 2001). This cardioprotection may involve both chaperone-mediated prevention of Sarcoplasmic Reticulum (SR) protein aggregation and calcium-binding-dependent restoration of Ca^2+^ homeostasis (Csermely et al., 1998; Nicchitta, 1998; Aridor and Balch, 1999).

### 3.4 Potential protective role of HSPD and HSPE (HSP60 and HSP10) in AF

The mitochondrial HSP60-HSP10 chaperonin system, where HSP10’s mobile loop (residues 25–40) binds HSP60 to modulate its ATPase activity (Höhfeld and Hartl, 1994), is essential for maintaining mitochondrial proteostasis (Ferenčić et al., 2020). Upon mitochondrial damage, the levels of both proteins exhibit a significant increase, thereby serving as a biomarker for mitochondrial stress.

This chaperpne system is ATP-dependent and exerts its protective effect via their regulation of electron transport chain (ETC) complex (Lin et al., 2001). From a structural perspective, HSP60 has a central hydrophobic cavity, providing an isolated environment for both *de novo* protein folding and matrix protein refolding. Moreover, at the molecular level, HSP60 prevents cardiomyocyte apoptotic death through reducing the release of cytochrome c and the activation of caspase-3.

**FIGURE 2:**
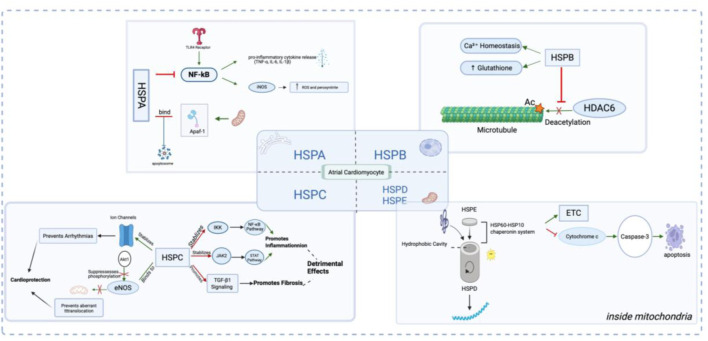
Illustration of the Role and Mechanism of Heat Shock Proteins (HSPs) in Atrial Myocytes. In response to atrial fibrillation (AF)-related stressors such as oxidative stress and inflammation, the HSP Family—including HSPA, HSPB, HSPC, and HSPD/E—exerts protective (or in some cases, detrimental) effects on atrial cardiomyocytes through a multitude of molecular mechanisms.

Historically, technological constraints led to considerable discrepancies between peripheral blood test results and findings from local cardiac tissue analyses. However, recent methodological advances have identified HSP60 as a promising biomarker. Current research is now utilizing ^18^F-fluorodeoxyglucose (FDG) positron emission tomography (PET) imaging to evaluate the role of HSP60 in cardiac inflammatory processes (Glaudemans et al., 2013). The research team led by Bi-Xi Chen enrolled 83 AF patients (43 with persistent AF and 40 with paroxysmal AF) and measured the FDG uptake activity of epicardial adipose tissue (EAT) using PET/CT. Comparative analyses were conducted before and after radiofrequency catheter ablation (RFCA). The Spearman correlation analysis showed that the extent of HSP60 reduction was significantly linked to the decrease in EAT activity, suggesting that HSP60 may play a role in alleviating cardiac inflammation following RFCA (Chen et al., 2021) (Figures 2, Table 1).

**TABLE 1:** Summary of the main numbers of HSP family and matching medications.

Family	Principal member	Relationship with AF	Functional properties in AF	Matching drugs	Drug structure	MOA
HSPA	HSP70	Higher in persistent AF patients compared to controls Lower in AF patients after RFCA.	suppresses autophagic activity and inflammation, stabilizes proteostasis	Inducer: TRC051384	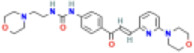	inhibits HDAC6, increases histone acetylation to indirectly activate HSF1 and enhance Hsp70 expression
				Inducer: tubastatin A	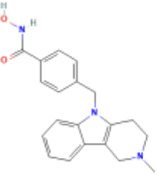	disrupts the HSF1-HSP90 complex, thereby releases HSF1 and enables it to bind to HSE, which subsequently activates Hsp70 transcription
HSPB	HSP27	Higher in paroxysmal AF patients than in persistent AF patients Elevated in patients with AF recurrence after pulmonary vein isolation (PVI)	safeguards microtubule integrity and reduces oxidative stress	Inducer: GGA	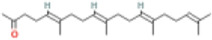	competes with endogenous geranyl groups to promote physiological RhoA activation, enhances the binding of HSF1 to the HSE in the promotor region of HSP genes
HSPC	HSP90	Elevated in patients with chronic AF.	stabilizes ion channel proteins and inhibits inflammation	Inhibitor: 17-AAG (Tanespimycin)	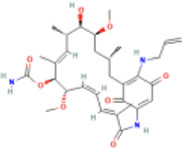	inhibit HSP90 expression by HSP90/FKBP5/HIF-1α/NCX1 signaling pathway
HSPD/HSPE	HSP60/HSP10	Reduction linked to decreased cardiac inflammation following RFCA.	prevents apoptotic death	No inducer or inhibitor for AF targeting HSP60/10		

Abbreviation: MOA, Mechanism Of Action; RFCA, radiofrequency ablation).

## 4 Research progress of HSPs inducers

In response to inflammation, oxidative stress, and disrupted proteostasis, cells activate HSR, leading to the upregulation of HSPs. The central regulator of the HSR is the Heat Shock Factor 1 (HSF1). Whereas, under normal physiological conditions, HSF1 is a monomer and mainly present in the cytosol. Under stress conditions, this conserved transcription factor is activated through trimerization and phosphorylation, after which it translocates to the nucleus and upregulates HSP genes by binding to heat shock response elements (HSEs) (Barna et al., 2018). Studies indicate that in short-duration AF, the HSR is activated, while it diminishes over time when AF persists (Brundel et al., 2006b). It has been recognized that AF-induced proteostasis derailment and subsequent electropathology is rooted in exhaustion of HSPs. Therefore, securing HSP levels at an adequate level, for example, by treatment with HSP inducers, may limit the expansion of the AF substrate during paroxysmal and short-term AF (Chang et al., 2013).

Currently, geranylgeranylacetone (GGA) stands as the most promising compound for the pharmacological induction of HSPs, which has been widely used in clinical practice as a treatment for ulcer in the digestive system. In recent years, GGA has been studied as a HSPs inducer, especially HSP27 and HSP70. GGA is a well-established drug that effectively boosts HSPB1 production, making it valuable for therapeutic applications targeting cellular stress responses (van Marion et al., 2020; Waddingham et al., 2023). GGA exerts its induced function through activating HSF-1 (van Marion et al., 2019). GGA may compete with endogenous geranyl groups, which could lead to inhibition of physiological RhoA activation, resulting in enhanced binding of HSF1 to the HSE in the promotor region of HSP genes (van Wijk et al., 2021). Therefore, GGA may induce the expression of HSPs by activating HSF-1 and promoting the binding of HSF1 to the HSE in the promotor region of HSP genes (Li et al., 2013).

However, although GGA has a protective effect on AF, its poor physical and chemical properties, including limited hydrophobicity and solubility, may adversely affect its application in AF. Due to the hydrophobicity of GGA, its distribution pattern of intestinal mucosa hinders its systemic availability, so it may be necessary to increase the dose to treat patients with AF. To address these limitations, researchers have developed multiple GGA derivatives with improved physicochemical properties. Denise et al.'s study synthesized 81 derivatives based on the molecular structure of GGA by shortening carbon chains, introducing hydrogen bond donors/acceptors, and replacing the central ketone group (with oxime, amide, pyrazole, etc.), with 7 compounds (including GGA*-59, LogP = 3.77) showing better HSP induction than GGA (van Marion et al., 2019). Hu et al. demonstrate that both HSP inducer GGA-59 and recombinant HSPB1 enhance recovery from tachypacing-induced structural remodeling and contractile dysfunction in HL-1 cardiomyocytes. Mechanistically, GGA-59 upregulates HSPB1 expression, inhibits HDAC6 activity and restores the expression of contractile proteins and microtubules after tachypacing (Hu et al., 2019; van Marion et al., 2019; van Wijk et al., 2021). GGA derivatives (especially GGA*-59) significantly enhance HSP-inducing capacity by optimizing physicochemical properties, effectively preventing and reversing myocardial dysfunction in experimental atrial fibrillation. However, these compounds remain at the experimental stage and have not yet been translated to clinical applications. The pharmacokinetics of GGA and its derivatives should be further investigated in in vivo models to assess their bioavailability.

The clinical application prospects of GGA are highly promising. Experiments conducted by Masao et al. demonstrated that GGA pretreatment significantly suppresses atrial conduction abnormalities and atrial fibrillation caused by myocardial ischemia by increasing HSP70 expression (Sakabe et al., 2008). Furthermore, studies have shown that 30%–50% of patients undergoing elective cardiothoracic surgery develop postoperative atrial fibrillation (Dobrev et al., 2019). Denise et al.'s randomized controlled trial (RCT) demonstrated that in 26 coronary artery bypass grafting (CABG) patients, just 3 days of GGA treatment (400 mg/day) significantly upregulated HSPB1 and HSPA1 expression in atrial tissue (P < 0.05) while enhancing HSPB1’s specific localization to myofilaments (P = 0.042). Notably, this short-term GGA intervention effectively elevated HSP levels in human atrial tissue, providing compelling theoretical evidence for GGA’s clinical application in preventing postoperative atrial fibrillation (PoAF) (van Marion et al., 2020). The GENIALITY (S Ramos et al., 2025) trial is the first randomized, double-blind, placebo-controlled study evaluating GGA for postoperative atrial fibrillation (PoAF) prevention. A total of 146 patients were randomly assigned in a 1:1 ratio to receive either 300 mg/day GGA or placebo, administered from 5 days before surgery until 3 days post-operation. PoAF incidence was assessed via continuous Holter monitoring, while HSP levels and atrial remodeling biomarkers were analyzed in collected samples. The clinical efficacy and pharmacological mechanisms of GGA can be comprehensively evaluated through these key indicators. Currently, the trial is still ongoing, and final results have not yet been published. As a single-center Phase II study with a limited sample size, its conclusions will require further validation through larger multicenter trials to assess its clinical applicability. If the findings are positive, GGA could emerge as a novel preventive strategy for PoAF and provide critical evidence for HSP-targeted therapies.

While GGA demonstrates promising HSP-inducing capacity and cardioprotection in experimental studies, its effects on hard clinical endpoints - particularly stroke prevention and long-term rhythm control - await confirmation in rigorous clinical trials (Brundel et al., 2006b; Sakabe et al., 2008). Notably, GGA is relatively contraindicated in certain clinical scenarios. GGA is primarily metabolized by the liver. In patients with hepatic impairment, dose adjustment and enhanced monitoring may be required. Additionally, concomitant use with drugs sharing the same metabolic pathway may lead to drug accumulation and adverse effects. Prolonged administration of teprenone may induce various adverse drug reactions, including but not limited to headache, cutaneous rash, constipation, abdominal distension, diarrhea, nausea, and epigastric pain, potentially limiting its therapeutic utility in certain clinical settings (Zheng et al., 2024) (Table 2).

**TABLE 2:** Summary of current clinical studies on heat shock proteins in atrial fibrillation.

Research title	N	Method	Endpoint	Key findings	Limitations
GENIALITY	146	assign 146 patients to receive either 300 mg/day GGA or placebo from 5 days pre-surgery to 3 days post-operation	PoAF incidence	trial is ongoing	1. Limited sample size 2. Single-center design 3. Phase II trial only 3.No dose-gradient comparison 4. No hard clinical endpoints
Bi-Xi Chen’s research	83	analyze FDG-PET/CT-quantified epicardial adipose tissue activity and HSP60 levelsn in AF patients before and after ablation	Correlation between HSP60 reduction and EAT activity decrease post-RFCA	HSP60 reduction correlated with decreased EAT activity (p < 0.05), suggesting its anti-inflammatory role post-ablation	1. Small sample size 2. Observational design 3. Short-term follow-up 4. No control group 5. EAT activity measurement variability suspected
Jelena Kornej’s research	67	measure serum and intracellular HSP70/anti-HSP70 levels via ELISA in AF patients before/after catheter ablation	Correlation between serum/intracellular HSP70 levels and AF recurrence risk post-ablation	Persistent AF showed elevated anti-HSP70, while intracellular HSP70 predicted lower recurrence	1. Small sample size 2. Single-center design 3. Short-term follow-up

In addition to the classic GGA, novel inducers TRC051384 and Tubastatin A have recently garnered significant attention. Both compounds primarily promote Hsp70 expression by modulating the activity of HSF1. TRC051384 likely exerts its effects by disrupting the HSF1-HSP90 complex, thereby releasing HSF1 and enabling it to bind to HSE, which subsequently activates Hsp70 transcription. Tubastatin A, as a selective HDAC6 inhibitor, increases histone acetylation to indirectly activate HSF1 and enhance Hsp70 expression (Kornej et al., 2013).

Additionally, the decreased levels of HSP C and HSP D/E are significantly associated with a reduced risk of atrial fibrillation onset (Huang et al., 2025; Chen et al., 2021). 17-AAG (Tanespimycin) is a specific HSP90 inhibitor that significantly reduces both the stability and transcriptional activity of the transcription factor HIF-1α (hypoxia-inducible factor-1α) by blocking its competitive binding with FKBP5 (FK506-binding protein 5). This mechanism ultimately leads to downregulation of NCX1 (sodium-calcium exchanger 1) expression, thereby improving calcium homeostasis in cardiomyocytes and effectively reducing the risk of atrial fibrillation (AF) onset. These findings provide an important theoretical foundation for developing novel antiarrhythmic drugs targeting the HSP90/FKBP5/HIF-1α/NCX1 signaling pathway (Wang et al., 2023) (Table 3).

**TABLE 3:** Translational status of HSP-based strategies for atrial fibrillation.

Strategy	Mechanism	Clinical readiness level	Major hurdles and rationale for ‘not ready’ status
HSPA as a Biomarker	In preclinical models, HSPA acts as a key cytoprotective chaperone by suppressing NF-κB-mediated inflammation, reducing ROS, and preserving protein homeostasis (Szyller et al., 2022; Kumar et al., 2021; Shi et al., 2023; Bellini et al., 2017)	Exploratory-Observational Jelena Kornej’s research found that only intracellular (not serum) HSP70 elevation post-ablation was associated with reduced AF recurrence. Persistent AF patients had higher baseline anti-HSP70 antibodies (Kornej et al., 2013; Mandal et al., 2005)	Invasive sampling was required for predictive value
HSPB as a Biomarker	*In vitro*, HSPB1 safeguards microtubule integrity by inhibiting HDAC6 (Hu et al., 2019) and reduces oxidative stress by increasing glutathione levels (Leite et al., 2016)	Exploratory-Observational Atrial tissue expression of HSP27 is higher in paroxysmal vs. persistent AF (Brundel et al., 2006b). Serum HSP27 levels were found to be elevated in patients with post-ablation AF recurrence (Marion et al., 2020; Hu et al., 2012)	Major discrepancy between tissue-level expression and circulating serum levels (Marion et al., 2020; van Marion et al., 2021)
Therapeutic HSP Induction	In animal/cell models, HSP inducers (like GGA) can upregulate HSP70/27, restore the microtubule network, reverse contractile dysfunction from tachypacing (Hu et al., 2019), and suppress ischemia-induced AF (Sakabe et al., 2008)	Early-Phase Clinical Trials A small RCT showed that oral GGA increased HSP expression in human atrial tissue of CABG patients (van Marion et al., 2020). A Phase II trial (referred to as GENIALITY in the text) is ongoing to assess GGA for preventing postoperative AF (PoAF) (S Ramos et al., 2025)	The primary compound, GGA, has poor solubility and bioavailability. Its derivatives (e.g., GGA*-59) are still in the experimental stage (van Marion et al., 2019). The clinical efficacy on hard endpoints for AF is unproven, awaiting trial results (S Ramos et al., 2025)
HSPC Inhibition	Preclinically, HSP90 inhibition can reduce inflammation by blocking NF-κB/STAT pathways (Huang et al., 2025) and improve cardiomyocyte calcium homeostasis by downregulating NCX1 expression via the HIF-1α pathway (Wang et al., 2023)	Preclinical 17-AAG have garnered significant attention as HSP90 inhibitors (Wang et al., 2023)	Lack of clinical data to support safety or efficacy in AF patients
	As a key mitochondrial chaperonin system, HSP60/10 is essential for mitochondrial proteostasis (Ferenčić et al., 2020). It prevents apoptosis and its levels increase under mitochondrial stress, making it a logical biomarker (Lin et al., 2001)	Exploratory (Imaging-based) A clinical study used 18F-FDG PET/CT to show that a reduction in serum HSP60 levels correlated with decreased epicardial adipose tissue (EAT) inflammatory activity after ablation (Glaudemans et al., 2013; Chen et al., 2021)	Absence of direct measurement of cardiac HSP60 (Lin et al., 2001; Glaudemans et al., 2013)

## 5 Discussion

AF remains a significant clinical challenge due to its complex pathophysiology, limited treatment efficacy, and high recurrence rates. This review highlights the emerging role of HSPs in AF pathogenesis, emphasizing their cardioprotective effects through anti-inflammatory, antioxidant, and proteostasis-preserving mechanisms. The evidence suggests that HSPs—particularly HSPA (HSP70) and HSPB (small HSPs)—play critical roles in mitigating atrial remodeling, stabilizing ion channels, and reducing oxidative stress, thereby potentially delaying AF progression.

Previous researches have investigated alterations in different HSP subtypes during AF, examining their correlation with AF onset and progression. HSPs showed potential as both diagnostic biomarkers for early AF detection and prognostic indicators for tracking disease advancement and severity (Rafaqat et al., 2023). Furthermore, the pharmacological induction of HSPs through GGA or similar agents represents a novel therapeutic strategy for AF, bridging the translational gap between preclinical models and clinical applications.

Although previous studies suggested that serum HSP levels (particularly HSP27) may have predictive value for AF (Marion et al., 2020; Rafaqat et al., 2023), Denise et al.'s study arrived at a different conclusion through systematic measurements. The research found that serum HSP levels (including HSPB1, HSPA1, HSPB7, and HSPD1) lacked clinical value in predicting postoperative atrial fibrillation (PoAF). However, the study revealed that the expression patterns of specific HSPs in atrial tissue were closely associated with AF progression and recurrence risk. Notably, patients with persistent AF showed significantly elevated HSPD1 levels in the right atrial appendage (RAA), while those with postoperative AF recurrence exhibited markedly higher HSPA1 and HSPA5 levels in the RAA. These findings suggest that tissue-specific HSPs may serve as potential biomarkers for AF staging and prognostic evaluation (van Marion et al., 2021). In summary, systemic factors such as inflammatory responses and oxidative stress may confound circulating HSP levels, resulting in imperfect correlation with myocardial stress severity. Future research should prioritize the development of cardiac-specific HSP detection methodologies to enhance the precision and clinical translatability of HSP-directed therapies. Currently, certain clinical applications have been implemented. The use of ^18^F-FDG PET/CT to link EAT inflammation with HSP60 levels is a pioneering step in this direction (Glaudemans et al., 2013).

Currently, the clinical development of HSP inducers is progressing rapidly, with multiple clinical trials being accelerated, demonstrating their potential therapeutic value in the field of atrial fibrillation treatment. Critically, to maximize their clinical translational value, the key lies in establishing a precise patient stratification system based on existing evidence, thereby ensuring these novel targeted therapies are accurately applied to specific patient populations most likely to benefit. Compared to patients with paroxysmal atrial fibrillation, those with persistent AF typically exhibit significantly lower HSPB1 expression levels, making them a more responsive target population for HSP inducer therapy. Research has found an inverse correlation between HSPB1 levels and the severity of AF. During AF progression, HSPB1 levels initially increase but subsequently decline, with significantly lower levels observed in persistent AF patients. This reduction compromises myocardial structure and disrupts cardiac electrical activity. Based on these findings, future research should focus on establishing HSP expression profiling criteria for AF patients, developing highly sensitive HSPB1 detection technologies, and conducting targeted clinical trials for HSP-deficient subgroups to advance precision therapy for AF (van Marion et al., 2019). Moreover, emerging evidence suggests that both AF pathophysiology and HSP levels exhibit demographic variations, particularly in terms of sex and age differences. Current research has conclusively demonstrated significant demographic variations in both the pathophysiological characteristics of AF and the expression levels of HSPs, with these differences primarily manifesting across gender and age dimensions. From a pathogenic perspective, female AF patients exhibit distinct clinical features: the incidence of non-pulmonary vein triggers is significantly higher than in male patients (Watanab et al., 2021). When looking at different age demographics, the elderly patients predominantly present with more severe atrial fibrosis (Aguiar et al., 2019), and the younger patients may derive preferential benefit from HSP-targeted therapies due to underlying mutation-derived ion channelopathies (e.g., TMEM168 mutations) linked to HSP dysfunction (Nguyen et al., 2021). These population-specific differences may be associated with the specificity of HSP regulation: studies indicate that estrogen may exert protective effects by upregulating the expression of HSPs (Shen et al., 2017). Conversely, the expression levels of HSPs show a marked decline during cellular senescence (Hebishy et al., 2023). These demographic-dependent variations in HSP expression provide a theoretical foundation for the precise application of HSP inducers in future therapeutic strategies. Future studies should validate these differential factors and further investigate additional stratification variables, including genetic and epigenetic determinants, to better realize their clinical translational potential (Table 4).

**TABLE 4:** Characteristics of atrial fibrillation patients stratified by type, age and gender.

Characteristics	Type of AF	Age		Gender
		Old	Young	
Manifestation	Persistent AF has reduced HSPB1 vs. paroxysmal AF, enhancing HSP inducer response	Atrial fibrosis is more severe	Benefit preferentially from mutation-derived ion channelopathies	Female AF patients show higher non-PV trigger rates than males

## 6 Conclusion

AF persists as a formidable clinical challenge, with its intricate molecular mechanisms requiring integrated diagnostic and therapeutic strategies. Novel targeted therapies like HSP inducers, including GGA and its derivatives—which may upregulate HSP expression by activating HSF-1 and enhancing its binding to HSEs in HSP gene promoter regions—could represent a paradigm shift in AF treatment. Continued translational research is essential to elucidate the remaining pathophysiological mysteries and optimize clinical outcomes for AF patients.

## References

[B1] Aguiar C. M. Gawdat K. Legere S. Marshall J. Hassan A. Kienesberger P. C. (2019). Fibrosis independent atrial fibrillation in older patients is driven by substrate leukocyte infiltration: diagnostic and prognostic implications to patients undergoing cardiac surgery. J. Transl. Med. 17 (1), 413. 10.1186/s12967-019-02162-5 31822289 PMC6905054

[B2] Allende M. Molina E. Guruceaga E. Tamayo I. González-Porras J. R. Gonzalez-López T. J. (2016). Hsp70 protects from stroke in atrial fibrillation patients by preventing thrombosis without increased bleeding risk. Cardiovasc. Res. 110 (3), 309–318. 10.1093/cvr/cvw049 26976620

[B3] Aridor M. Balch W. E. (1999). Integration of endoplasmic reticulum signaling in health and disease. Nat. Med. 5 (7), 745–751. 10.1038/10466 10395318

[B4] Aschar-Sobbi R. Izaddoustdar F. Korogyi A. S. Wang Q. Farman G. P. Yang F. (2015). Increased atrial arrhythmia susceptibility induced by intense endurance exercise in mice requires TNFα. Nat. Commun. 6, 6018. 10.1038/ncomms7018 25598495 PMC4661059

[B5] Attia A. Muthukumarasamy K. M. Al-U'Datt D. G. F. Hiram R. (2025). Relevance of targeting oxidative stress, inflammatory, and pro-resolution mechanisms in the prevention and management of postoperative atrial fibrillation. Antioxidants Basel, Switz. 14 (4), 414. 10.3390/antiox14040414 PMC12023940 40298654

[B6] Ayzenberg O. Swissa M. Shlezinger T. Bloch S. Katzir I. Chodick G. (2023). Atrial fibrillation ablation success rate - a retrospective multicenter study. Curr. problems Cardiol. 48 (8), 101161. 10.1016/j.cpcardiol.2022.101161 35245600

[B7] Barna J. Csermely P. Vellai T. (2018). Roles of heat shock factor 1 beyond the heat shock response. Cell. Mol. life Sci. CMLS 75 (16), 2897–2916. 10.1007/s00018-018-2836-6 29774376 PMC11105406

[B8] Bellini S. Barutta F. Mastrocola R. Imperatore L. Bruno G. Gruden G. (2017). Heat shock proteins in vascular diabetic complications: review and future perspective. Int. J. Mol. Sci. 18 (12), 2709. 10.3390/ijms18122709 29240668 PMC5751310

[B9] Brundel B. J. Henning R. H. Ke L. van Gelder I. C. Crijns H. J. Kampinga H. H. (2006b). Heat shock protein upregulation protects against pacing-induced myolysis in HL-1 atrial myocytes and in human atrial fibrillation. J. Mol. Cell. Cardiol. 41 (3), 555–562. 10.1016/j.yjmcc.2006.06.068 16876820

[B10] Brundel B. J. Shiroshita-Takeshita A. Qi X. Yeh Y. H. Chartier D. van Gelder I. C. (2006a). Induction of heat shock response protects the heart against atrial fibrillation. Circulation Res. 99 (12), 1394–1402. 10.1161/01.RES.0000252323.83137.fe 17110598

[B11] Chang S. L. Chen Y. C. Hsu C. P. Kao Y. H. Lin Y. K. Lai Y. J. (2013). Heat shock protein inducer modifies arrhythmogenic substrate and inhibits atrial fibrillation in the failing heart. Int. J. Cardiol. 168 (4), 4019–4026. 10.1016/j.ijcard.2013.06.072 23871620

[B12] Chen B. X. Xie B. Zhou Y. Shi L. Wang Y. Zeng L. (2021). Association of serum biomarkers and cardiac inflammation in patients with atrial fibrillation: identification by positron emission tomography. Front. Cardiovasc. Med. 8, 735082. 10.3389/fcvm.2021.735082 34712708 PMC8546267

[B13] Csermely P. Schnaider T. Soti C. Prohászka Z. Nardai G. (1998). The 90-kDa molecular chaperone family: structure, function, and clinical applications. A comprehensive review. Pharmacol. Ther. 79 (2), 129–168. 10.1016/s0163-7258(98)00013-8 9749880

[B14] Currie R. W. (1988). Protein synthesis in perfused rat hearts after *in vivo* hyperthermia and *in vitro* cold ischemia. Biochem. cell Biol. = Biochimie Biol. Cell. 66 (1), 13–19. 10.1139/o88-002 3370140

[B15] Dobrev D. Aguilar M. Heijman J. Guichard J. B. Nattel S. (2019). Postoperative atrial fibrillation: mechanisms, manifestations and management. Nat. Rev. Cardiol. 16 (7), 417–436. 10.1038/s41569-019-0166-5 30792496

[B16] Ferenčić A. Cuculić D. Stemberga V. Šešo B. Arbanas S. Jakovac H. (2020). Left ventricular hypertrophy is associated with overexpression of HSP60, TLR2, and TLR4 in the myocardium. Scand. J. Clin. laboratory investigation 80 (3), 236–246. 10.1080/00365513.2020.1725977 32057259

[B17] Gao G. Dudley S. C. Jr (2009). Redox regulation, NF-kappaB, and atrial fibrillation. Antioxidants redox Signal. 11 (9), 2265–2277. 10.1089/ars.2009.2595 PMC2819799 19309257

[B18] Glaudemans A. W. de Vries E. F. Galli F. Dierckx R. A. Slart R. H. Signore A. (2013). The use of (18)F-FDG-PET/CT for diagnosis and treatment monitoring of inflammatory and infectious diseases. Clin. Dev. Immunol. 2013, 623036. 10.1155/2013/623036 24027590 PMC3763592

[B19] Hazra J. Vijayakumar A. Mahapatra N. R. (2023). Emerging role of heat shock proteins in cardiovascular diseases. Adv. protein Chem. Struct. Biol. 134, 271–306. 10.1016/bs.apcsb.2022.10.008 36858739

[B20] He Y. Hara H. Núñez G. (2016). Mechanism and regulation of NLRP3 inflammasome activation. Trends Biochem. Sci. 41 (12), 1012–1021. 10.1016/j.tibs.2016.09.002 27669650 PMC5123939

[B21] Hebishy M. Shintouo C. M. Dufait I. Debacq-Chainiaux F. Bautmans I. Njemini R. (2023). Heat shock proteins and cellular senescence in humans: a systematic review. Archives gerontology geriatrics 113, 105057. 10.1016/j.archger.2023.105057 37207540

[B22] Henning R. H. Brundel B. J. J. M. (2017). Proteostasis in cardiac health and disease. Nat. Rev. Cardiol. 14 (11), 637–653. 10.1038/nrcardio.2017.89 28660894

[B23] Hindricks G. Potpara T. Dagres N. Arbelo E. Bax J. J. Blomström-Lundqvist C. (2021). 2020 ESC guidelines for the diagnosis and management of atrial fibrillation developed in collaboration with the european association for cardio-thoracic surgery (EACTS): the task force for the diagnosis and management of atrial fibrillation of the european society of cardiology (ESC) developed with the special contribution of the european heart rhythm association (EHRA) of the ESC. Eur. heart J. 42 (5), 373–498. 10.1093/eurheartj/ehaa612 32860505

[B24] Höhfeld J. Hartl F. U. (1994). Role of the chaperonin cofactor Hsp10 in protein folding and sorting in yeast mitochondria. J. cell Biol. 126 (2), 305–315. 10.1083/jcb.126.2.305 7913473 PMC2200036

[B25] Hu X. Li J. van Marion D. M. S. Zhang D. Brundel B. J. J. M. (2019). Heat shock protein inducer GGA*-59 reverses contractile and structural remodeling *via* restoration of the microtubule network in experimental atrial fibrillation. J. Mol. Cell. Cardiol. 134, 86–97. 10.1016/j.yjmcc.2019.07.006 31302117

[B26] Hu Y. F. Yeh H. I. Tsao H. M. Tai C. T. Lin Y. J. Chang S. L. (2012). Electrophysiological correlation and prognostic impact of heat shock protein 27 in atrial fibrillation. Circulation. Arrhythmia Electrophysiol. 5 (2), 334–340. 10.1161/CIRCEP.111.965996 22354927

[B27] Hu Z. Ding L. Yao Y. (2023). Atrial fibrillation: mechanism and clinical management. Chin. Med. J. 136 (22), 2668–2676. 10.1097/CM9.0000000000002906 37914663 PMC10684204

[B28] Huang J. R. Chen L. Li C. Q. (2025). Ethoxyquin mediates lung fibrosis and cellular immunity in BLM-CIA mice by inhibiting HSP90. Adv. Clin. Exp. Med. official organ Wroclaw Med. Univ. 34 (2), 211–225. 10.17219/acem/186365 39283681

[B29] Ihara K. Sasano T. (2022). Role of inflammation in the pathogenesis of atrial fibrillation. Front. physiology 13, 862164. 10.3389/fphys.2022.862164 PMC9047861 35492601

[B30] Iwai C. Li P. Kurata Y. Hoshikawa Y. Morikawa K. Maharani N. (2013). Hsp90 prevents interaction between CHIP and HERG proteins to facilitate maturation of wild-type and mutant HERG proteins. Cardiovasc. Res. 100 (3), 520–528. 10.1093/cvr/cvt200 23963841

[B31] Kahlhofer J. Leon S. Teis D. Schmidt O. (2021). The α-arrestin family of ubiquitin ligase adaptors links metabolism with selective endocytosis. Biol. cell 113 (4), 183–219. 10.1111/boc.202000137 33314196

[B32] Kampinga H. H. Hageman J. Vos M. J. Kubota H. Tanguay R. M. Bruford E. A. (2009). Guidelines for the nomenclature of the human heat shock proteins. Cell stress chaperones 14 (1), 105–111. 10.1007/s12192-008-0068-7 18663603 PMC2673902

[B33] Ke L. Meijering R. A. Hoogstra-Berends F. Mackovicova K. Vos M. J. Van Gelder I. C. (2011). HSPB1, HSPB6, HSPB7 and HSPB8 protect against RhoA GTPase-induced remodeling in tachypaced atrial myocytes. PloS one 6 (6), e20395. 10.1371/journal.pone.0020395 21731611 PMC3123278

[B34] Kirmanoglou K. Hannekum A. Schäfler A. E. (2004). Expression of mortalin in patients with chronic atrial fibrillation. Basic Res. Cardiol. 99 (6), 404–408. 10.1007/s00395-004-0477-4 15309412

[B35] Kishore P. Collinet A. C. T. Brundel B. J. J. M. (2023). Prevention of atrial fibrillation: putting proteostasis derailment back on track. J. Clin. Med. 12 (13), 4352. 10.3390/jcm12134352 37445387 PMC10342900

[B36] Kornej J. Börschel C. S. Benjamin E. J. Schnabel R. B. (2020). Epidemiology of atrial fibrillation in the 21st century: novel methods and new insights. Circulation Res. 127 (1), 4–20. 10.1161/CIRCRESAHA.120.316340 32716709 PMC7577553

[B37] Kornej J. Reinhardt C. Kosiuk J. Arya A. Hindricks G. Adams V. (2013). Response of circulating heat shock protein 70 and anti-heat shock protein 70 antibodies to catheter ablation of atrial fibrillation. J. Transl. Med. 11, 49. 10.1186/1479-5876-11-49 23432758 PMC3599085

[B38] Kumar S. Gupta E. Gupta N. Kaushik S. Srivastava V. K. Kumar S. (2021). Functional role of iNOS-Rac2 interaction in neutrophil extracellular traps (NETs) induced cytotoxicity in sepsis. Clin. chimica acta; Int. J. Clin. Chem. 513, 43–49. 10.1016/j.cca.2020.12.004 33309799

[B39] Lakin R. Polidovitch N. Yang S. Parikh M. Liu X. Debi R. (2023). Cardiomyocyte and endothelial cells play distinct roles in the tumour necrosis factor (TNF)-Dependent atrial responses and increased atrial fibrillation vulnerability induced by endurance exercise training in mice. Cardiovasc. Res. 119 (16), 2607–2622. 10.1093/cvr/cvad144 37713664 PMC10730243

[B40] Leite J. S. Raizel R. Hypólito T. M. Rosa T. D. Cruzat V. F. Tirapegui J. (2016). l-glutamine and l-alanine supplementation increase glutamine-glutathione axis and muscle HSP-27 in rats trained using a progressive high-intensity resistance exercise. Appl. physiology, Nutr. metabolism = Physiologie appliquee, Nutr. metabolisme 41 (8), 842–849. 10.1139/apnm-2016-0049 27447686

[B41] Li N. Brundel B. J. J. M. (2020). Inflammasomes and proteostasis novel molecular mechanisms associated with atrial fibrillation. Circulation Res. 127 (1), 73–90. 10.1161/CIRCRESAHA.119.316364 32717176 PMC7388703

[B42] Li Y. Li J. Cui L. Lai Y. Yao Y. Zhang Y. (2013). Inhibitory effect of atorvastatin on AGE-Induced HCAEC apoptosis by upregulating HSF-1 protein. Int. J. Biol. Macromol. 57, 259–264. 10.1016/j.ijbiomac.2013.03.035 23511056

[B43] Lin K. M. Lin B. Lian I. Y. Mestril R. Scheffler I. E. Dillmann W. H. (2001). Combined and individual mitochondrial HSP60 and HSP10 expression in cardiac myocytes protects mitochondrial function and prevents apoptotic cell deaths induced by simulated ischemia-reoxygenation. Circulation 103 (13), 1787–1792. 10.1161/01.cir.103.13.1787 11282911

[B44] Mandal K. Torsney E. Poloniecki J. Camm A. J. Xu Q. Jahangiri M. (2005). Association of high intracellular, but not serum, heat shock protein 70 with postoperative atrial fibrillation. Ann. Thorac. Surg. 79 (3), 865–871. 10.1016/j.athoracsur.2004.08.018 15734396

[B45] Marion D. M. S. V. Lanters E. A. H. Ramos K. S. Li J. Wiersma M. Baks-Te Bulte L. (2020). Evaluating serum heat shock protein levels as novel biomarkers for atrial fibrillation. Cells 9 (9), 2105. 10.3390/cells9092105 32947824 PMC7564530

[B46] Masuda D. Nakanishi I. Ohkubo K. Ito H. Matsumoto K. I. Ichikawa H. (2024). Mitochondria play essential roles in intracellular protection against oxidative stress-which molecules among the ROS generated in the mitochondria can escape the mitochondria and contribute to signal activation in cytosol? Biomolecules 14 (1), 128. 10.3390/biom14010128 38275757 PMC10813015

[B47] Nguyen L. K. C. Shimizu A. Soh J. E. C. Komeno M. Sato A. Ogita H. (2021). Transmembrane protein 168 mutation reduces cardiomyocyte cell surface expression of Nav1.5 through αB-crystallin intracellular dynamics. J. Biochem. 170 (5), 577–585. 10.1093/jb/mvab066 34086898

[B48] Nicchitta C. V. (1998). Biochemical, cell biological and immunological issues surrounding the endoplasmic reticulum chaperone GRP94/gp96. Curr. Opin. Immunol. 10 (1), 103–109. 10.1016/s0952-7915(98)80039-3 9523119

[B49] Pandey S. N. Agrawal N. Moglad E. Priya G. P. Srivastava M. Chennakesavulu K. (2025). CHIP and aging: a key regulator of proteostasis and cellular senescence. Biogerontology 26 (3), 104. 10.1007/s10522-025-10247-6 40323531

[B50] Rafaqat S. Rafaqat S. Rafaqat S. (2023). The role of major biomarkers of stress in atrial fibrillation: a literature review. J. innovations cardiac rhythm Manag. 14 (2), 5355–5364. 10.19102/icrm.2023.14025 PMC9983621 36874560

[B51] Ren M. Li X. Hao L. Zhong J. (2015). Role of tumor necrosis factor alpha in the pathogenesis of atrial fibrillation: a novel potential therapeutic target? Ann. Med. 47 (4), 316–324. 10.3109/07853890.2015.1042030 25982799

[B52] Romanucci M. Della Salda L. (2015). Oxidative stress and protein quality control systems in the aged canine brain as a model for human neurodegenerative disorders. Oxidative Med. Cell. Longev. 2015, 940131. 10.1155/2015/940131 PMC4442305 26078824

[B53] Sakabe M. Shiroshita-Takeshita A. Maguy A. Brundel B. J. Fujiki A. Inoue H. (2008). Effects of a heat shock protein inducer on the atrial fibrillation substrate caused by acute atrial ischaemia. Cardiovasc. Res. 78 (1), 63–70. 10.1093/cvr/cvn019 18238941

[B54] Schäuble N. Lang S. Jung M. Cappel S. Schorr S. Ulucan Ö. (2012). BiP-mediated closing of the Sec61 channel limits Ca2+ leakage from the ER. EMBO J. 31 (15), 3282–3296. 10.1038/emboj.2012.189 22796945 PMC3411083

[B55] Schwabl S. Teis D. (2022). Protein quality control at the golgi. Curr. Opin. cell Biol. 75, 102074. 10.1016/j.ceb.2022.02.008 35364487

[B56] Scott L. Jr Li N. Dobrev D. (2019). Role of inflammatory signaling in atrial fibrillation. Int. J. Cardiol. 287, 195–200. 10.1016/j.ijcard.2018.10.020 30316645 PMC6447485

[B57] Shan Q. Ma F. Wei J. Li H. Ma H. Sun P. (2020). Physiological functions of heat shock proteins. Curr. protein peptide Sci. 21 (8), 751–760. 10.2174/1389203720666191111113726 31713482

[B58] Shen H. H. Huang S. Y. Cheng P. Y. Chu Y. J. Chen S. Y. Lam K. K. (2017). Involvement of HSP70 and HO-1 in the protective effects of raloxifene on multiple organ dysfunction syndrome by endotoxemia in ovariectomized rats. Menopause (New York, N.Y.) 24 (8), 959–969. 10.1097/GME.0000000000000864 28350760

[B59] Shi T. Wang G. Peng J. Chen M. (2023). Loss of MD1 promotes inflammatory and apoptotic atrial remodelling in diabetic cardiomyopathy by activating the TLR4/NF-κB signalling pathway. Pharmacology 108 (4), 311–320. 10.1159/000530081 37231994

[B60] Singh M. K. Shin Y. Ju S. Han S. Choe W. Yoon K. S. (2024). Heat shock response and heat shock proteins: current understanding and future opportunities in human diseases. Int. J. Mol. Sci. 25 (8), 4209. 10.3390/ijms25084209 38673794 PMC11050489

[B61] Sirish P. Diloretto D. A. Thai P. N. Chiamvimonvat N. (2022). The critical roles of proteostasis and endoplasmic reticulum stress in atrial fibrillation. Front. physiology 12, 793171. 10.3389/fphys.2021.793171 PMC8764384 35058801

[B62] S Ramos K. Nassiri S. Wijdeveld L. F. J. van der Palen R. L. Kuipers M. F. Hills M. T. (2025). Geranylgeranylacetone as prevention for postoperative atrial fibrillation (GENIALITY). Cardiovasc. drugs Ther. 10.1007/s10557-025-07693-2 PMC12717209 40227474

[B63] Sun X. Moreno Caceres S. Yegambaram M. Lu Q. Pokharel M. D. Boehme J. T. (2024). The mitochondrial redistribution of ENOS is regulated by AKT1 and dimer status. Nitric oxide Biol. Chem. 152, 90–100. 10.1016/j.niox.2024.09.009 PMC12068231 39332480

[B64] Szyller J. Kozakiewicz M. Siermontowski P. Kaczerska D. (2022). Oxidative stress, HSP70/HSP90 and eNOS/iNOS serum levels in professional divers during hyperbaric exposition. Antioxidants Basel, Switz. 11 (5), 1008. 10.3390/antiox11051008 PMC9137907 35624872

[B65] Tashiro S. Miyake H. Rokutan K. (2018). Role of geranylgeranylacetone as non-toxic HSP70 inducer in liver surgery: clinical application. J. hepato-biliary-pancreatic Sci. 25 (5), 269–274. 10.1002/jhbp.549 29658197

[B66] van Gorp P. R. R. Trines S. A. Pijnappels D. A. de Vries A. A. F. (2020). Multicellular *in vitro* models of cardiac arrhythmias: focus on atrial fibrillation. Front. Cardiovasc. Med. 7, 43. 10.3389/fcvm.2020.00043 32296716 PMC7138102

[B67] van Marion D. M. Hu X. Zhang D. Hoogstra-Berends F. Seerden J. G. Loen L. (2019). Screening of novel HSP-Inducing compounds to conserve cardiomyocyte function in experimental atrial fibrillation. Drug Des. Dev. Ther. 13, 345–364. 10.2147/DDDT.S176924 PMC6342224 30705583

[B68] van Marion D. M. S. Dorsch L. Hoogstra-Berends F. Kakuchaya T. Bockeria L. de Groot N. M. S. (2020). Oral geranylgeranylacetone treatment increases heat shock protein expression in human atrial tissue. Heart rhythm. 17 (1), 115–122. 10.1016/j.hrthm.2019.07.010 31302249

[B69] van Marion D. M. S. Ramos K. S. Lanters E. A. H. Bulte L. B. Bogers A. J. J. C. de Groot N. M. S. (2021). Atrial heat shock protein levels are associated with early postoperative and persistence of atrial fibrillation. Heart rhythm. 18 (10), 1790–1798. 10.1016/j.hrthm.2021.06.1194 34186247

[B70] van Wijk S. W. Ramos K. S. Brundel B. J. J. M. (2021). Cardioprotective role of heat shock proteins in atrial fibrillation: from mechanism of action to therapeutic and diagnostic target. Int. J. Mol. Sci. 22 (1), 442. 10.3390/ijms22010442 33466228 PMC7795054

[B71] Vitadello M. Ausma J. Borgers M. Gambino A. Casarotto D. C. Gorza L. (2001). Increased myocardial GRP94 amounts during sustained atrial fibrillation: a protective response? Circulation 103 (17), 2201–2206. 10.1161/01.cir.103.17.2201 11331263

[B72] Vyas V. Hunter R. J. Longhi M. P. Finlay M. C. (2020). Inflammation and adiposity: new frontiers in atrial fibrillation. Europace 22 (11), 1609–1618. 10.1093/europace/euaa214 33006596

[B73] Waddingham M. T. Sequeira V. Kuster D. W. D. Dal Canto E. Handoko M. L. de Man F. S. (2023). Geranylgeranylacetone reduces cardiomyocyte stiffness and attenuates diastolic dysfunction in a rat model of cardiometabolic syndrome. Physiol. Rep. 11 (22), e15788. 10.14814/phy2.15788 37985159 PMC10659935

[B74] Wang J. Lee J. Liem D. Ping P. (2017). HSPA5 gene encoding Hsp70 chaperone BiP in the endoplasmic reticulum. Gene 618, 14–23. 10.1016/j.gene.2017.03.005 28286085 PMC5632570

[B75] Wang X. Song J. Yuan Y. Li L. Abu-Taha I. Heijman J. (2023). Downregulation of FKBP5 promotes atrial arrhythmogenesis. Circulation Res. 133 (1), e1–e16. 10.1161/CIRCRESAHA.122.322213 37154033 PMC10330339

[B76] Watanabe K. Nitta J. Inaba O. Sato A. Inamura Y. Kato N. (2021). Predictors of non-pulmonary vein foci in paroxysmal atrial fibrillation. J. interventional cardiac Electrophysiol. Int. J. Arrhythm. pacing 61 (1), 71–78. 10.1007/s10840-020-00779-x 32468323

[B77] Wei Y. Zhuang Y. Zhang Y. Luo L. Yu B. Zeng J. (2024). Role of heat shock protein 70 in silibinin-induced apoptosis in bladder cancer. J. Cancer 15 (1), 79–89. 10.7150/jca.88668 38164275 PMC10751677

[B78] Yamada N. Matsushima-Nishiwaki R. Kobayashi K. Takahata S. Toyoda H. Kumada T. (2021). Cellular functions of small heat shock proteins (HSPB) in hepatocellular carcinoma. Curr. Mol. Med. 21 (10), 872–887. 10.2174/1573405617666210204211252 33563195

[B79] Yang X. Zhang W. Wen X. Bulinski P. J. Chomchai D. A. Arines F. M. (2020). TORC1 regulates vacuole membrane composition through ubiquitin- and ESCRT-Dependent microautophagy. J. cell Biol. 219 (3), e201902127. 10.1083/jcb.201902127 32045480 PMC7055007

[B80] Zakkar M. Ascione R. James A. F. Angelini G. D. Suleiman M. S. (2015). Inflammation, oxidative stress and postoperative atrial fibrillation in cardiac surgery. Pharmacol. Ther. 154, 13–20. 10.1016/j.pharmthera.2015.06.009 26116810

[B81] Zhang D. Ke L. Mackovicova K. Van Der Want J. J. Sibon O. C. Tanguay R. M. (2011). Effects of different small HSPB members on contractile dysfunction and structural changes in a *Drosophila melanogaster* model for atrial fibrillation. J. Mol. Cell. Cardiol. 51 (3), 381–389. 10.1016/j.yjmcc.2011.06.008 21745477

[B82] Zhang D. Wu C. T. Qi X. Meijering R. A. Hoogstra-Berends F. Tadevosyan A. (2014). Activation of histone deacetylase-6 induces contractile dysfunction through derailment of α-tubulin proteostasis in experimental and human atrial fibrillation. Circulation 129 (3), 346–358. 10.1161/CIRCULATIONAHA.113.005300 24146251

[B83] Zhang Y. Chen X. Zhao Y. Ponnusamy M. Liu Y. (2017). The role of ubiquitin proteasomal system and autophagy-lysosome pathway in alzheimer's disease. Rev. Neurosci. 28 (8), 861–868. 10.1515/revneuro-2017-0013 28704199

[B84] Zheng Y. Song J. Huang L. Chen G. Ning N. Huang Q. (2024). WeiNaiAn capsule attenuates intestinal mucosal injury and regulates gut microbiome in indomethacin-induced rat. Int. J. Biochem. cell Biol. 173, 106609. 10.1016/j.biocel.2024.106609 38880193

